# Gastrectomy for cancer beyond life expectancy. A comprehensive analysis of oncological gastric surgery in Germany between 2008 and 2018

**DOI:** 10.3389/fonc.2022.1032443

**Published:** 2022-11-30

**Authors:** Maximilian Berlet, Marie-Christin Weber, Philipp-Alexander Neumann, Helmut Friess, Daniel Reim

**Affiliations:** Department of Surgery, Klinikum rechts der Isar, Technical University of Munich, Munich, Germany

**Keywords:** elderly patients, gastrectomy, gastric cancer, esophageal cancer, clinical outcome, comorbidity, mortality

## Abstract

**Introduction:**

Major gastric surgery for distal esophageal and gastric cancer has a strong impact on the quality of life, morbidity, and mortality. Especially in elderly patients reaching their life expectancy, the responsible use and extent of gastrectomy are imperative to achieve a balance between harm and benefit. In the present study, the reimbursement database (German Diagnosis Related Groups (G-DRG) database) of the Statistical Office of the Federal Republic of Germany was queried to evaluate the morbidity and mortality of patients aged above or below 75 years following gastrectomy.

**Material and methods:**

All patients in Germany undergoing subtotal gastrectomy (ST), total gastrectomy (T), or gastrectomy combined with esophagectomy (TE) for gastric or distal esophageal cancer (International Statistical Classification of Diseases and Related Health Problems Version 10 (ICD-10) C15.2, C15.5, and C16.0–C16.9) between 2008 and 2018 were included. Intraoperative and postoperative complications as well as comorbidities, in-hospital mortality, and the extent of surgery were assessed by evaluating ICD-10 and operation and procedure key (Operationen- und Prozedurenschlüssel) codes.

**Results:**

A total of 67,389 patients underwent oncologic gastric resection in Germany between 2008 and 2018. In total, 21,794 patients received ST, 41,825 received T, and 3,466 received TE, respectively. In 304 cases, the combinations of these, in fact, mutually exclusive procedures were encoded. The proportion of patients aged 75 years or older was 51.4% (n = 11,207) for ST, 32.6% (n = 13,617) for T, and 28.1% (n = 973) for TE. The in-hospital mortality of elderly patients was significantly increased in all three groups. (p < 0.0001) General complications such as respiratory failure (p = 0.0054), acute renal failure (p < 0.0001), acute myocardial failure (p < 0.0001), and the need for resuscitation (ST/T: p < 0.0001/TE: p = 0.0218) were significantly increased after any kind of gastrectomy. Roux-en Y was the most commonly applied reconstruction technique in both young and elderly patients. Regarding lymphadenectomy, systematic D2 dissection was performed less frequently in older patients than in the younger collective in the case of ST and T as well as D3 dissection. Peritonectomy and hyperthermic intraperitoneal chemotherapy were uncommon in elderly patients alongside ST and T compared to younger patients (p < 0.0001).

**Conclusion:**

The clinical outcome of major oncological gastric surgery is highly dependent on a patient’s age. The elderly show a tremendously increased likelihood of in-hospital mortality and morbidity.

## Introduction

Oncologic resection for gastric cancer by either partial or total gastrectomy is the main pillar of curative treatment aside from multimodal therapies in advanced stages, which may be a critical matter from numerous aspects. On one hand, an oncologically radical approach is important in order to achieve R0 resection (1), but, on the other hand, gastrectomy is correlated to a high mortality rate of up to 20% (2). In particular, age-related aspects regarding surgery for gastric cancer have not been adequately investigated yet. Thus, most studies only focus on surgical technique and cancer specifications and do not cover the entire collective of elderly patients requiring therapy for gastric cancer (3, 4). Articles related to elderly gastrectomy patients usually address the best surgical technique in terms of minimally invasive and robotic-assisted surgery (5–7). With regard to stratification by age, several aspects are of particular interest. For example, perioperative mortality and the probability of other postoperative complications must be weighed against current life expectancy without surgery (8). Recent study collectives are mostly small in number. In addition, the results being published may be biased due to the fact that these studies are mostly reporting on patient cohorts from specialized treatment centers not representing common clinical nationwide practice. Therefore, a systematic analysis of large case numbers is urgently needed to evaluate the influence of age on the surgical outcome related to major gastric surgery, especially when life expectancy is reached. The aim of the present study was to evaluate the clinical outcomes of patients aged beyond the average life expectancy undergoing surgery for gastric cancer in a population-based study using a structured query of the German Diagnosis Related Groups (G-DRG) database of the German Statistical Office (DESTATIS). The complete database is accessible only for selected researchers and contains all the diagnoses and medical procedures of inpatients treated in German hospitals, which were encoded according to the International Classification of Diseases version 10 with the German modification (ICD-10-GM) and the German operation and procedure key (‘Operationen- und Prozedurenschlüssel’, OPS) (9).

## Material and methods

All patients with gastric and distal esophageal cancer (ICD C15.2, C15.5, and C16.0–C16.9) receiving major gastric surgery in terms of subtotal gastrectomy (ST, OPS 5-435, 5-436), total gastrectomy (T, OPS 5-437), and total gastrectomy with esophageal resection (TE, OPS 5-438) in Germany between 2008 and 2018 were included. The parameters queried comprised comorbidity, reconstruction technique, the extent of lymphadenectomy (LAD), adjunctive therapy and organ resection, intraoperative and postoperative adverse events, and perioperative mortality. These factors were then analyzed for age dependency by setting a cutoff at 75 years. Patients younger than this age were assigned to group L75 (‘less than 75 years’), and older patients were assigned to group G75 (‘greater or equal to 75 years’). Intraoperative and postoperative complications were defined according to the international consensus on complications after gastrectomy for cancer (10). The source code for the query of the G-DRG database was created in the SAS programming language, as required by DESTATIS (9). The same program code was executed separately for each year of interest. Diagnoses and complications were defined using the most appropriate ICD-10 and OPS codes available (see the supplement for details). Statistical analysis was then performed using R statistical software version 3.6 without additional packages (11). To calculate significance, the Wilcoxon rank sum test was used for the Charlson comorbidity scores and the chi-square test and Fisher’s exact test were applied to nominal scaled parameters. In case of multiple testing, Bonferroni correction was used to adjust p-values. The particular statistical tests applied to the data and the absolute subgroup sizes are depicted in each table and figure. Relative frequencies are given for mortality, complications, the reconstruction technique, LAD, and adjunctive therapy. The Charlson index for comorbidity is reported as the mean and standard deviation for each collective and year. The significance level was set at 5%.

## Results

A total of 67,389 patients with gastric or distal esophageal cancer underwent major gastric resection in Germany during the observation period (ST: 21,794/T: 41,825/TE: 3,466/combinations of OPS codes for ST, T, or TE: 304 cases). The proportion of patients with an age of 75 years or more (G75) was 51.4% (n = 11,207) for ST, 32.6% (n = 13,617) for T, and 26.9% for TE (n = 973).

For the analysis of comorbidity, the Charlson comorbidity index in its classic version was calculated for both collectives (12). For each individual year and each type of gastric resection, there was a significantly higher score found in the elderly group (**Table 1**).

**Table 1:** Charlson comorbidity score of patients undergoing major gastric surgery in Germany between 2008 and 2018.

Group		Parameter	2008	2009	2010	2011	2012	2013	2014	2015	2016	2017	2018	p-value
**<75**	**ST**	**Mean**	**4.77**	**4.89**	**5.15**	**4.98**	**5.11**	**5.32**	**5.44**	**5.5**	**5.22**	**5.4**	**5.58**	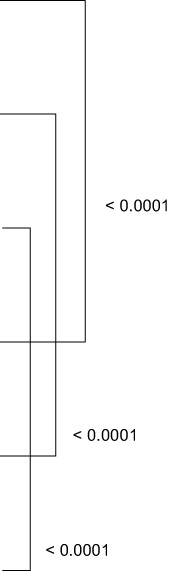
		sd	3.2	3.23	3.4	3.34	3.37	3.45	3.46	3.57	3.62	3.49	3.56	
		n	1,144	1,095	1,012	969	962	946	945	946	848	869	851	
	**T**	**Mean**	**4.77**	**4.9**	**5.17**	**5.13**	**5.2**	**5.46**	**5.49**	**5.64**	**5.56**	**5.79**	**5.64**	
		sd	3.32	3.34	3.45	3.45	3.44	3.53	3.51	3.57	3.5	3.54	3.48	
		n	2,887	2,774	2,661	2,598	2,475	2,395	2,843	2,701	2,382	2,314	2,178	
	**TE**	**Mean**	**4.5**	**5.41**	**4.94**	**5.38**	**5.23**	**5.86**	**5.74**	**6.17**	**5.73**	**6.05**	**5.88**	
		sd	3.27	3.41	3.3	3.16	3.56	3.71	3.41	3.59	3.45	3.56	3.51	
		n	140	179	135	148	150	152	214	194	403	406	372	
**≥75**	**ST**	**Mean**	**6.76**	**7.16**	**7.49**	**7.21**	**7.46**	**7.54**	**7.71**	**7.96**	**7.9**	**7.87**	**7.96**	
		sd	3.07	3.24	3.34	3.31	3.41	3.31	3.48	3.61	3.47	3.64	3.57	
		n	1,167	1,089	1,016	988	987	984	1,029	1,057	945	992	953	
	**T**	**Mean**	**6.89**	**6.99**	**7.39**	**7.37**	**7.44**	**7.79**	**7.86**	**7.79**	**8.12**	**7.97**	**8**	
		sd	3.26	3.22	3.43	3.42	3.4	3.52	3.56	3.51	3.62	3.55	3.57	
		n	1,205	1,208	1,108	1,197	1,208	1,237	1,380	1,385	1,297	1,228	1,164	
	**TE**	**Mean**	**7.44**	**6.81**	**7.21**	**7.24**	**8**	**7.88**	**7.14**	**7.82**	**7.88**	**8.25**	**8.47**	
		sd	3.21	3.38	3.11	3.37	3.6	3.48	3.1	4.1	3.61	3.53	3.67
		n	57	54	56	54	48	59	71	79	164	173	158

< 75: patients, younger than 75 years, ≥ 75: patients with an age of 75 years or older, ST, subtotal gastrectomy, T, total gastrectomy, TE, combined total gastrectomy and esophageal resection, mean, mean of the Charlson comorbidity index, sd, standard deviation of the Charlson comorbidity index, n, number of patients in the particular group; the Wilcoxon rank sum test with Bonferroni adjustment was used for statistical testing with a level of significance set at 5%. The difference between L75 and G75 was significant for each single year under study.

The bold values represent the mean values.

**Figure 1:**
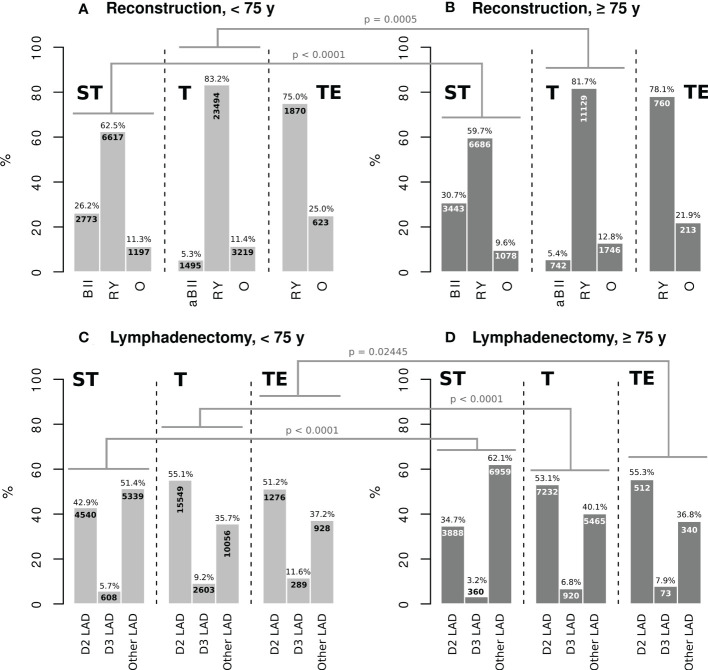
ST: subtotal gastrectomy, T: total gastrectomy, TE: gastrectomy combined with esophagectomy, BII: Billroth II reconstruction, aBII reconstruction analogue to Billroth II, RY: Roux en-Y reconstruction, O: other reconstruction technique, D2 LAD: straight D2 lymphadenectomy, D3 LAD: straight D3 lymphadenectomy, Other LAD: extent of lymphadenectomy other than straight systematic D2 or D3, Chi-square test with Bonferroni adjustment was applied for statistical testing. The significance level was set at 5%.

The pattern of reconstruction techniques, in terms of the use of Billroth II (BII), analog to Billroth II (aBII), Roux en Y-like (RY), or other (O) reconstruction, was almost the same in both age groups with slight differences regarding modified techniques. Nevertheless, the number of BII reconstructions was higher in elderly patients undergoing ST (30.7 vs. 26.2%). Following all gastric resection types, RY was the most frequently used technique in both groups (**Figures 1A, B**). Regarding LAD, systematic D2 LAD was the most frequently used procedure after total gastrectomy and gastrectomy combined with esophagectomy in patients <75 years and ≥75 years. D3 dissection was performed less commonly in the G75 collective (ST: 3.2 vs. 5.7%/T: 6.8 vs. 9.2%/TE: 7.9 vs. 11.6%) After subtotal resection, LAD strategies other than straight systematic D2 or D3 LAD were used in 51.4% (L75) and 62.1% (G75) including partly D2 or D3 dissection, respectively (**Figures 1C, D**).

While the type of reconstruction did not differ substantially between the two age groups, the extent of further therapy and additional organ resection appeared to be markedly divergent. Less aggressive approaches were observed for pancreatic resection after subtotal and total gastrectomy (ST: 1.3 vs. 2.3%, OR 0.58, CI_95%_ 0.46–0.71, p < 0.0001/T: 3.6 vs. 4.5%, OR 0.80, CI_95%_ 0.72–0.89, p = 0.0005). Partial or total adrenalectomy was less frequently performed in the G75 group alongside total gastrectomy (0.4 vs. 0.9%, OR 0.48, CI_95%_ 0.35–0.64, p < 0.0001). In contrast, there was no significant difference between the two groups in terms of splenectomy for subtotal and total gastrectomy or combined gastrectomy and esophagectomy. Most extensive methods, such as peritonectomy and hyperthermic intraperitoneal chemotherapy (HIPEC), were used rarely in elderly patients in association with all three types of gastric surgery (**Table 2**).

**Table 2:** Adjunctive therapy and organ resection alongside oncological gastric resection in elderly patients in Germany between 2008 and 2018.

Adjunctivetherapy	Subtotal gastrectomy (ST)							Total gastrectomy (T)							Total gastrectomy and esophageal resection (TE)						
	< 75 y		≥ 75 y					< 75 y		≥ 75 y					< 75 y		≥ 75 y				
	n	%	n	%	OR	CI_95%_	p-value	n	%	n	%	OR	CI_95%_	p-value	n	%	n	%	OR	CI_95%_	p-value
Pancreatic resection	**239** (10,587)	2.3%	**147** (11,207)	1.3%	0.58	0.46–0.71	**<0.0001**	**1,270** (28,208)	4.5%	**494** (13,617)	3.6%	0.80	0.72–0.89	**0.0005**	**147** (2,493)	5.9%	**41** (863)	4.8%	0.79	0.54–1.14	1.0000
Liver resection	**609** (10,587)	5.8%	**570** (11,207)	5.1%	0.88	0.78–1.00	0.6508	**1,791** (28,208)	6.3%	**771** (13,617)	5.7%	0.89	0.81–1.00	0.1282	**179** (2,493)	8.4%	**77** (919)	7.2%	1.18	0.88–1.57	1.0000
Splenectomy	**206** (10,587)	1.9%	**267** (11,207)	2.4%	1.23	1.00–1.49	0.6040	**2,898** (28,208)	10.3%	**1,412** (13,617)	10.4%	1.01	0.94–1.08	1.0000	**317** (2,493)	12.7%	**119** (973)	12.2%	0.96	0.76–1.20	1.0000
Peritonectomy	**108** (9,443)	1.1%	**23** (6,044)	0.4%	0.33	0.20–0.52	**<0.0001**	**741** (28,208)	2.6%	**118** (12,412)	0.95%	0.36	0.29–0.43	**< 0.0001**	**75** (2,279)	3.2%	**0** (161)	0.0%	–	–	0.1801
HIPEC	**58** (9,443)	1.1%	**0** (9,087)	0.0%	–	–	**<0.0001**	**568** (25,321)	2.2%	**21** (11,204)	0.19%	0.08	0.05–0.13	**< 0.0001**	**33** (1,316)	2.5%	**0** (471)	0.0%	–	–	**0.0014**
Colon resection	**398** (10,587)	3.8%	**434** (11,207)	3.9%	1.03	0.89–1.90	1.0000	**1,224** (28,208)	4.3%	**623** (13,617)	4.6%	1.06	0.95–1.67	1.0000	**114** (2,493)	4.6%	**36** (812)	4.4%	0.97	0.64–1.43	1.0000
Adrenalectomy	**21** (5,523)	0.38%	**16** (5,988)	0.27%	0.70	0.34–1.41	1.0000	**252** (28,208)	0.9%	**53** (12,409)	0.4%	0.48	0.35–0.64	**< 0.0001**	**42** (2,149)	2.0%	**3** (592)	0.5%	0.26	0.05–0.80	0.2136

ST, subtotal gastrectomy; T, total gastrectomy; TE, total gastrectomy in combination with esophageal resection; HIPEC, hyperthermic intraperitoneal chemotherapy; <75 y, patients younger than 75 years; ≥75 y, patients at an age of 75 years or more; n, absolute number of patients; the numbers in brackets delineate the overall collective after indexing by the Statistical Office; OR, odds ratio; CI95%, 95% confidence interval; Fisher’s exact test with the Bonferroni adjustment of p-values was used for statistical testing. The level of significance was set at 5%.

The bold values denote statistical significance at P <0.05 level.

General and surgical complications were subgrouped into intraoperative and postoperative adverse events according to the international consensus of complications after gastrectomy for cancer (10). The intraoperative course in terms of unintended injury to anatomic structures such as solid organs and blood vessels, during ST and T was slightly increased in elderly patients. (ST: 1.7 vs. 1.2%, OR 1.43, CI_95%_ 1.14–1.81, p = 0.0173/T: 2.2 vs. 1.7%, OR 1.28, CI_95%_ 1.10–1.48, p = 0.0109) Intraoperative bleeding and the need for interruption of surgery were not impacted by age (**Table 3A**).

Table 3Intraoperative and postoperative general and surgical complications in elderly patients undergoing oncological gastric resection in Germany between 2008 and 2018.

**Table:** 

A Intraoperative	Subtotal gastrectomy (ST)							Total gastrectomy (T)							Total gastrectomy and esophageal resection (TE)						
	< 75 y		≥ 75 y					< 75 y		≥ 75 y					< 75 y		≥ 75 y				
	n	%	n	%	OR	CI_95%_	p-value	n	%	n	%	OR	CI_95%_	p-value	n	%	n	%	OR	CI_95%_	p-value
Unintended intraoperative damage to vessels or organs	**128** (10,587)	1.2%	**193** (11,207)	1.7%	1.43	1.14–1.81	**0.0173**	**486** (28,208)	1.7%	**298** (13,617)	2.2%	1.28	1.10–1.48	**0.0109**	**50** (2,174)	2.3%	**17** (467)	3.6%	1.60	0.85–2.86	0.9378
Intraoperative bleeding	**474** (10,587)	4.5%	**543** (11,207)	4.8%	1.09	0.96–1.24	1.0000	**1,236** (28,208)	4.4%	**677** (13,617)	5.0%	1.14	1.04–1.26	0.0676	**139** (2,341)	5.9%	**54** (812)	6.7%	1.13	0.80–1.57	1.0000
Interruption of the planned procedure	**30** (5,488)	0.5%	**35** (7,193)	0.49%	0.89	0.53–1.50	1.0000	**87** (28,208)	0.3%	**43** (9,987)	0.4%	1.40	0.95–2.04	0.7965	**0** (924)	0.0%	**0** (450)	0.0%	–	–	–

B Postoperative general	Subtotal gastrectomy (ST)							Total gastrectomy (T)							Total gastrectomy and esophageal resection (TE)						
	<75 y		≥75 y					<75 y		≥75 y					<75 y		≥75 y				
	n	%	n	%	OR	CI_95%_	p-value	n	%	n	%	OR	CI_95%_	p-value	n	%	n	%	OR	CI_95%_	p-value
Apoplexy	**55** (8,656)	0.64%	**122** (11,207)	1.1%	1.72	1.24–2.41	**0.0275**	**106** (28,208)	0.38%	**99** (13,617)	0.73%	1.94	1.46–2.58	0.1081	**10** (1,399)	0.71%	**3** (738)	0.41%	0.57	0.09–2.21	1.0000
Need for resuscitation	**160** (10,587)	1.5%	**293** (11,207)	2.6%	1.75	1.44–2.14	**< 0.0001**	**479** (28,208)	1.7%	**457** (13,617)	3.4%	2.01	1.76–2.29	**< 0.0001**	**69** (2,341)	2.9%	**51** (919)	5.5%	1.93	1.30–2.84	**0.0218**
Myocardial infarction	**206** (10,587)	1.9%	**356** (11,207)	3.2%	1.65	1.39–1.98	**< 0.0001**	**629** (28,208)	2.2%	**535** (13,617)	3.9%	1.79	1.59–2.02	**< 0.0001**	**80** (2,493)	3.2%	**52** (919)	5.7%	1.81	1.23–2.21	0.0642
Cardiac dysrhythmia	**167** (10,587)	1.6%	**435** (11,207)	3.9%	2.52	2.10–3.04	**< 0.0001**	**571** (28,208)	2.0%	**544** (13,617)	4.0%	2.01	1.78–2.27	**< 0.0001**	**91** (2,493)	3.7%	**56** (902)	6.2%	1.75	1.22–2.49	0.0762
Acute myocardial failure	**219** (10,587)	2.1%	**714** (11,207)	6.4%	3.22	2.76–3.77	**< 0.0001**	**488** (28,208)	1.7%	**803** (13,617)	5.9%	3.56	3.17–4.00	**< 0.0001**	**44** (2,279)	1.9%	**66** (866)	7.6%	4.19	2.79–6.34	**< 0.0001**
Pulmonary embolism	**167** (10,587)	1.6%	**195** (11,207)	1.7%	1.10	0.89–1.37	1.0000	**523** (28,208)	1.9%	**261** (13,617)	1.9%	1.03	0.88–1.20	1.0000	**65** (2,070)	3.1%	**22** (733)	3.0%	0.95	0.55–1.58	1.0000
Respiratory failure	**1092** (10,587)	10.3%	**1,595** (11,207)	14.2%	1.44	1.32–1.57	**< 0.0001**	**3,407** (28,208)	12.1%	**2,420** (13,617)	17.8%	1.57	1.48–1.67	**< 0.0001**	**489** (2,493)	19.6%	**249** (973)	25.6%	1.41	1.17–1.68	**0.0054**
Need for tracheostomy	**231** (10,587)	2.2%	**273** (11,207)	2.4%	1.12	0.93–1.34	1.0000	**778** (28,208)	2.8%	**515** (13,617)	3.8%	1.39	1.23–1.55	**< 0.0001**	**128** (2,493)	5.1%	**65** (973)	6.7%	1.32	0.95–1.81	1.0000
Need for prolonged intubation	**29** (7,848)	0.37%	**27** (5,110)	0.53%	1.43	0.81–2.51	1.0000	**101** (25,813)	0.39%	**54** (9,915)	0.54%	1.39	0.98–1.96	1.0000	**17** (1,759)	0.97%	**3** (446)	0.67%	0.69	0.12–2.42	1.0000
Liver dysfunction	**466** (10,587)	4.4%	**548** (11,207)	4.9%	1.11	0.98–1.27	1.0000	**1,428** (28,208)	5.1%	**832** (13,617)	6.1%	1.22	1.11–1.33	**< 0.0001**	**159** (2,493)	6.4%	**53** (764)	6.9%	1.09	0.77–1.52	1.0000
Acute renal insufficiency	**530** (10,587)	5.0%	**1,077** (11,207)	9.6%	2.02	1.80–2.25	**< 0.0001**	**1,406** (28,208)	5.0%	**1,422** (13,617)	10.4%	2.22	2.06–2.40	**< 0.0001**	**198** (2,493)	7.9%	**131** (973)	13.5%	1.80	1.41–2.29	**0.0001**
Infection	**1,152** (10,587)	10.9%	**1,826** (11,207)	16.3%	1.59	1.47–1.73	**< 0.0001**	**3124** (28,208)	11.1%	**2,157** (13,617)	15.8%	1.51	1.42–1.60	**< 0.0001**	**301** (2,493)	12.1%	**166** (973)	17.1%	1.50	1.21–1.85	**0.0058**

C gastrectomy (ST)	Total gastrectomy (T)							Total gastrectomy and esophageal resection (TE)						
	<75 y		≥75 y					<75 y		≥75 y					<75 y		≥75 y				
	**n**	**%**	**n**	**%**	**OR**	**CI_95%_**	**p-value**	**n**	**%**	**n**	**%**	**OR**	**CI_95%_**	**p-value**	**n**	**%**	**n**	**%**	**OR**	**CI_95%_**	**p-value**
Need for blood transfusion	**2,918** (8,348)	35.0%	**4,987** (8,951)	55.7%	2.34	2.20–2.49	**< 0.0001**	**8,563** (22,547)	38.0%	**6,323** (11,204)	56.4%	2.12	2.02–2.22	**< 0.0001**	**835** (2,174)	38.4%	**468** (862)	54.3	1.90	1.61–2.24	**< 0.0001**
Bowel obstruction	**184** (10,587)	1.7%	**218** (11,207)	1.9%	1.12	0.91–1.37	1.0000	**354** (28,208)	1.3%	**176** (13,617)	1.3%	1.03	0.85–1.24	1.0000	**20** (1,565)	1.3%	**10** (619)	1.6%	1.27	0.53–2.86	1.0000
Bowel perforation	**47** (9,443)	0.5%	**94** (11,207)	0.84%	1.69	1.17– 2.46	0.0882	**194** (28,208)	0.69%	**153** (13,617)	1.1%	1.64	1.32–2.04	**0.0002**	**11** (1,357)	0.81%	**0** (159)	0.0%	–	–	1.0000
Duodenal leak	**23** (6,660)	0.35%	**24** (6,239)	0.38%	1.11	0.60–2.07	1.0000	**22** (15,675)	0.14%	**25** (7,403)	0.34%	2.41	1.30–4.49	0.0826	**3** (1,312)	0.23%	**3** (914)	0.33%	1.44	0.19–10.7	1.0000
Anastomotic leakage^1^	**281** (5,405)	5.2%	**313** (5,960)	5.3%	1.01	0.85–1.19	1.0000	**1,090** (14,813)	7.4%	**756** (7,691)	9.8%	1.37	1.24–1.51	**< 0.0001**	**182** (1,741)	10.4%	**85** (704)	12.1%	1.18	0.88–1.56	1.0000
Pancreatic fistula	**563** (10,587)	5.3%	**559** (11,207)	5.0%	0.93	0.82–1.06	1.0000	**1,796** (28,208)	6.4%	**818** (13,617)	6.0%	0.94	0.86–1.02	1.0000	**193** (2,493)	7.7%	**64** (919)	7.0%	0.89	0.65–1.20	1.0000
Pancreatitis	**45** (8,679)	0.52%	**32** (8,279)	0.39%	0.74	0.45–1.20	1.0000	**115** (28,208)	0.41%	**66** (13,617)	0.48%	1.19	0.86–1.63	1.0000	**7** (1,392)	0.5%	**0** (667)	0.0%	–	–	1.0000
Need for abdominal drainage	**397** (10,587)	3.7%	**433** (11,207)	3.9%	1.03	0.89–1.19	1.0000	**1,144** (28,208)	4.0%	**619** (13,617)	4.5%	1.12	1.01–1.25	0.6243	**129** (2,493)	5.2%	**49** (737)	6.6%	1.31	0.90–1.85	1.0000
Impaired gastric emptying	**3** (3,876)	0.08%	**16** (6,144)	0.26%	3.37	0.96–18.07	1.0000	–	–	–	–	–	–	–	–	–	–	–	–	–	–
Other complications	**1,904** (10,587)	18.0%	**2183** (11,207)	19.5%	1.10	1.02–1.18	0.1473	**5,461** (28,208)	19.4%	**2,969** (13,617)	21.8%	1.16	1.10–1.22	**< 0.0001**	**576** (2,493)	23.1%	**233** (973)	23.9%	1.05	0.88–1.25	1.0000

ST, subtotal gastrectomy; T, total gastrectomy; TE, total gastrectomy in combination with esophageal resection; <75 y, patients younger than 75 years; ≥75 y, patients at an age of 75 years or more; n, absolute number of patients; the numbers in brackets delineate the overall collective after indexing by the Statistical Office; OR, odds ratio; CI_95%_, 95% confidence interval; ^1^The ICD-10-GM code for anastomotic leakage was just introduced in 2013; Fisher’s exact test with the Bonferroni adjustment of p-values was used for statistical testing. The level of significance was set at 5%.

The bold values denote statistical significance at P < 0.05 level.

Significant differences between the two groups, L75 and G75, were found in the postoperative course. General complications such as respiratory and acute renal failure, acute myocardial dysfunction, and the need for resuscitation were increased after subtotal and total gastrectomy in G75. In addition, elderly patients were significantly more susceptible to infections after all three types of gastric resection were studied (**Table 3B**).

Regarding specific surgical complications, the elderly had a significantly increased need for blood transfusions after each type of surgery. Furthermore, older patients showed an increased risk of bowel perforation (1.1 vs. 0.69%, OR 1.64, CI_95%_ 1.32–2.04, p < 0.0002) and anastomotic leakage (9.8 vs. 7.4%, OR 1.37, CI_95%_ 1.24–1.51, p < 0.0001) if they received total gastrectomy (**Table 3C**).

In-hospital mortality in elderly patients was higher after all three types of gastrectomy compared with the L75 group. (ST: 11.0 vs. 4.36%, OR 2.71, CI_95%_ 2.42–3.03, p < 0.0001/T: 11.9 vs. 4.23%, OR 3.05, CI_95%_ 2.82–3.30, p < 0.0001/TE: 13.9 vs. 5.86%, OR 2.59, CI_95%_ 1.99–3.35, p < 0.0001) (**Table 4**)

**Table 4:** In-hospital mortality following oncological gastric surgery in elderly patients in Germany between 2008 and 2018.

	< 75 y			≥ 75 y					
	n	In-hospital death	Mortality	n	In-hospital death	Mortality	OR	CI_95%_	p-value
**ST**	10,587	462	4.36%	11,207	1,233	11.0%	2.71	2.42–3.03	<0.0001
**T**	28,208	1,193	4.23%	13,617	1,618	11.9%	3.05	2.82–3.30	<0.0001
**TE**	2,493	146	5.86%	916	127	13.9%	2.59	1.99–3.35	<0.0001

ST, subtotal gastrectomy; T, total gastrectomy; TE, total gastrectomy in combination with esophageal resection; <75 y, patients younger than 75 years; ≥75 y, patients at an age of 75 years or more; n, absolute number of patients; OR, odds ratio; CI_95%_: 95% confidence interval, Fisher’s exact test with the Bonferroni adjustment of p-values was used for statistical testing. The level of significance was set at 5%.

## Discussion

The present study assessed differences in outcome after major oncologic gastric surgery among patients aged 75 years and older in Germany between 2008 and 2018 based on the G-DRG database of the German Federal Statistical Office (DESTATIS). Significant differences were found in particular with respect to postoperative morbidity and mortality.

Comorbidity was measured using the Charlson comorbidity index. The elderly collective scored significantly higher compared to younger patients stratified by each kind of gastric resection and each single year under study. Obviously, the difference is biased by the fact that the age of a particular patient is part of the calculation formula for the Charlson comorbidity index itself. An age between 80 and 89 years adds 4 points to the score, and an age of more than 90 years contributes to even 5 points, respectively. Nevertheless, the mean score suggests a 10-year mortality of 47% in the L75 group and 79% in the G75 collective solely based on comorbidity profile without considering the surgeries performed, indicating a certain vulnerability among the elderly group (12). For this reason, especially if life expectancy is reached, preoperative comorbidities and clinical circumstances must thoroughly be taken into account when planning major gastric resection for cancer in elderly patients.

The pattern of reconstruction techniques was rather similar in both groups. Merely after ST, there were more BII-like reconstructions performed in the elderly, and, after T, a slightly increased rate of reconstruction techniques ‘other than BII and RY’ was seen in this group. All in all, the age does not influence the choice of reconstruction technique fundamentally. Unfortunately, the current version of the OPS does not reflect the whole range of possible reconstruction techniques in detail. As case numbers in western Europe are not comparable to that in Asia, the implementation of new reconstruction approaches and their representation in the relevant coding systems are still hampered (13).

Regarding LAD, straight D2 and D3 LAD seems to be applied less frequently to elderly patients. Instead, other strategies like partial D2 or D3 LAD are more common in this group (see the supplement for the exact code definition used for the query). The fact that LAD is performed to an altered extent in elderly gastric cancer patients is already known and has been shown to be appropriate in Asian populations (14). The influence on the clinical outcome of adapted LAD in elderly patients in a western collective cannot fully be uncovered by the presented study. However, recent research suggests that standard D2 gastrectomy can safely be applied even to elderly patients (15, 16). Further research and clear recommendations are urgently needed on this field, as the presented data suggest, that D2 LAD seems to be applied hesitantly to patients older than 75 years, which may influence the oncologic outcome. Nonetheless, D2 dissection rates were surprisingly low although D2 LAD was adopted as a standard surgical procedure in the local guidelines. It may be speculated that D2 is either not performed according to the guideline recommendations or that the coding was not done appropriately. The influence of modified LAD on postoperative outcomes, therefore, cannot be finally evaluated in the setting of this analysis.

Another indicator of an adapted approach in elderly patients is the lower rate of additive organ resections such as pancreatic resection and peritonectomy. In addition, HIPEC is applied less frequently to the G75 collective. A less aggressive approach in gastric surgery for the elderly has been observed previously, as a reduction in the dimension of treatment may significantly improve the complication profile and should be considered in these patients (17). The data indicate an already-present clear consideration about the kind and extent of adjunctive surgery in daily clinical practice. Thus, pancreatic resection in the case of ST and T and adrenalectomy alongside T represent the only significantly altered organ resection approaches applied to elderly patients. The rates of colon and liver resection as well as splenectomy are not significantly divergent compared to younger patients.

Aside from a slightly increased rate of unintended damaging of blood vessels and organs, there appears to exist no significant influence of advanced age on the immediate intraoperative course. Bleeding during surgery and the interruption of the planned procedure are not impacted. Of course, a possibly explorative intent of a surgery cannot be deduced ultimately from the presented data. Even the postoperative surgical course seems only to be influenced by three particular aspects, namely, the necessity of blood transfusions, a higher rate of bowel perforation, and anastomotic leakage after total gastrectomy, aside from an increase in ‘other complications’ (see the supplement for code definition). However, we cannot derive the actual reason for a higher rate of transfusions from the data. As intraoperative bleeding seems not to be responsible, there may be other aspects like a decreased ability for compensation in the presence of low hemoglobin values among the elderly group. As already mentioned above in terms of comorbidity, a preoperative assessment of anemia and age-appropriate management could improve the outcome and avoid the extensive use of blood transfusions (18). Regarding the increased incidence of anastomotic leakage after total gastrectomy in the elderly, nutritional aspects and comorbidity may be important factors and further research is required to overcome this life-threatening adverse event (19).

Non-surgical complications like respiratory, renal or myocardial failure, and the need for cardio pulmonary resuscitation (CPR) in case of cardiac arrest are significantly increased in the elderly. Moreover, the distinct susceptibility to postoperative infections must be taken into account. However, there are preoperative screening tools available or currently under development addressing this problem; there is an urgent need for further improvement (20–23). In this sense, even an appropriate assessment of the mentioned postoperative complications and adverse events with clear recommendations would be helpful to minimize morbidity.

Regarding postoperative mortality, there was a more-than-doubled probability of in-hospital death in the elderly collective. The rates of 11%–13%, depending on the extent of resection, highlight once again the vulnerability of that group compared to younger patients and raise the question of whether less invasive surgery might be of advantage in the selected subgroups of this collective. However, a meta-analysis by Kong et al. did not show a difference regarding morbidity and mortality between T and ST without regard on age; the extent of resection seems to be relevant in elderly patients (24).

There exist several limitations in the presented study regarding data quality and the informative value. The necessity to avoid small group sizes in the query strategy for the G-DRG database to minimize the probability of indexing by the Statistical Office for secrecy reasons delimits the grade of detail, like the particular strategy of LAD in each subgroup. Furthermore, only morbidity and procedures operationalized within the ICD-10 and OPS are evaluable. For example, there exists no information about the histological subtype of a tumor if not explicitly defined in the particular code. Our intent to use the international consensus list of complications after gastrectomy could not be realized ultimately as the pieces of information cannot be derived from the DESTATIS database (10). Finally, the quality of data depends highly on the sincerity of the encoding personnel in the hospitals, and economical interests may bias the data to a certain degree. Aside from this, it may not be deducted from the data presented if patients died from aggressive or progressing tumor burden or the complication itself. This fact further limits the generalizability of the data presented.

All in all, the impact of age on the perioperative outcome of patients undergoing gastrectomy is still controversial and cannot be fully uncovered by the present data. Varying endpoints and cutoffs in recent studies further complicate a comprehensive overview of the underlying issues. For instance, a cutoff age of 45 years with the definition that patients older than 45 years are ‘elderly’ in an exemplary study by Cheng et al. in 2021 suggests that there exists no difference between young and older patients during and after gastrectomy regarding several complications contrarily to our results (25). On the other hand, there exist several publications that confirm our impression of a significant impact of age on the postoperative outcome after gastrectomy (8, 26). Many articles about elderly patients already indicate a general consideration of geriatric aspects in major gastric surgery as this group is obviously more susceptible to numerous complications. Nevertheless, further systematic investigation is mandatory as there do not exist valid and comprehensive recommendations regarding a reasonable balance between surgical extent and the oncological outcome in the western collectives of elderly patients with gastric cancer yet.

## Conclusion

The presented results demonstrate that the immediate outcome of major oncological gastric surgery depends highly on age aspects. Elderly patients have a tremendously increased likelihood of in-hospital morbidity and mortality, a fact that must be considered thoroughly when planning gastric resection. Nonetheless, the present data allow a real-life evaluation of all surgical gastric cancer cases in Germany and should be respected when counseling patients to decide for further therapeutic steps. Further research and new approaches to individualized geriatric surgery for gastrectomy are urgently needed in that sense.

## Data availability statement

The raw data supporting the conclusions of this article will be made available by the authors, without undue reservation.

## Author contributions

MB: Programming of the query to the Federal Statistical Office of Germany, data evaluation, statistical analysis, literature review, manuscript writing M-CW: data evaluation, statistical analysis, literature review, manuscript writing P-AN: data evaluation, statistical analysis, literature review, manuscript writing HF: data evaluation, statistical analysis, literature review, manuscript writing DR: data evaluation, statistical analysis, literature review, manuscript writing. All authors contributed to the article and approved the submitted version.

## Conflict of interest

The authors declare that the research was conducted in the absence of any commercial or financial relationships that could be construed as a potential conflict of interest.

## Publisher’s note

All claims expressed in this article are solely those of the authors and do not necessarily represent those of their affiliated organizations, or those of the publisher, the editors and the reviewers. Any product that may be evaluated in this article, or claim that may be made by its manufacturer, is not guaranteed or endorsed by the publisher.
