# Supplementary material for: Gastrectomy for cancer beyond life expectancy. A comprehensive analysis of oncological gastric surgery in Germany between 2008 and 2018
Source: Front Oncol. 2022 Nov 30;12:1032443. doi: 10.3389/fonc.2022.1032443 (PMC9747770; doi:10.3389/fonc.2022.1032443)
Supplement: Supplementary file 1 [file DataSheet_1.pdf]

Supplementary Material

Supplementary Figures

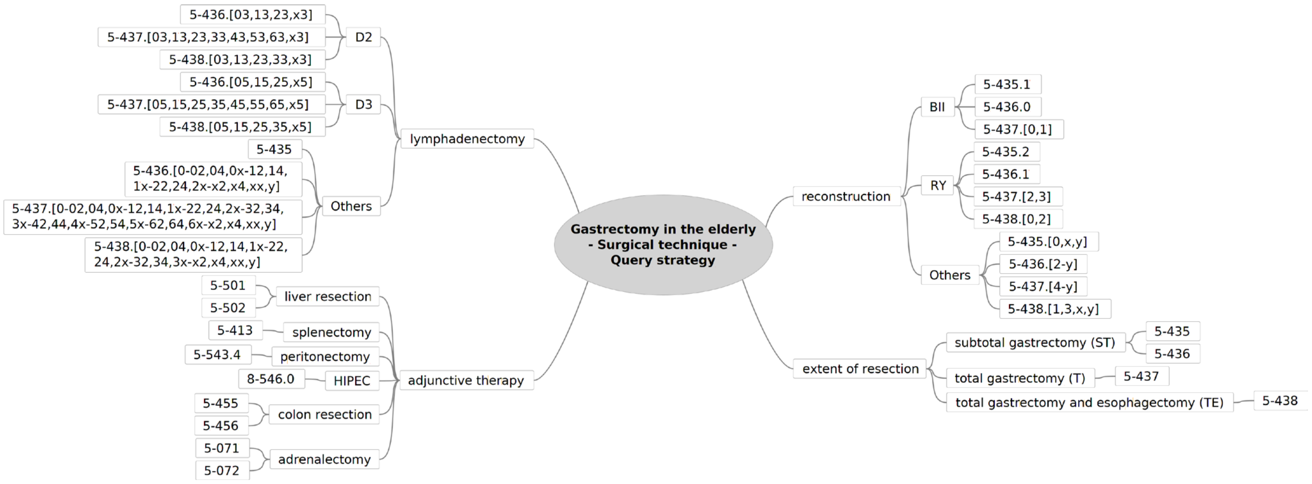

Supplementary figure 1: Query strategy for surgical extent, reconstruction, lymphadenectomy, and adjunctive therapy used for oncological gastric resection in Germany between 2008 and 2018, BII: Billroth II, RY: Roux en-Y, D2: D2 LAD, D3: D3 LAD

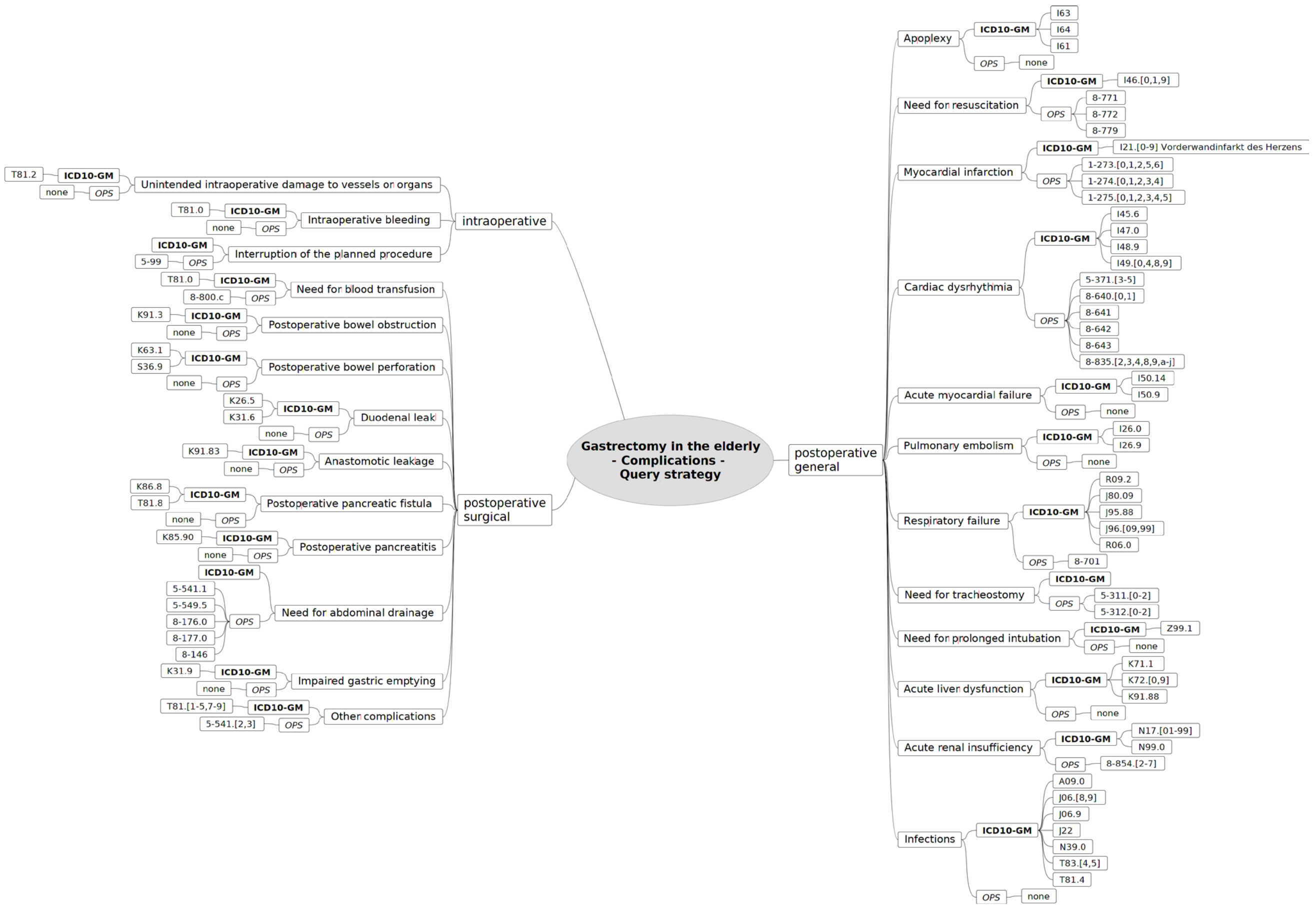

Supplementary figure 2: Query strategy for complications of oncological gastric resection in Germany between 2008 and 2018, ICD10-GM: International Classification of Diseases version 10 with the German modification, OPS: German operation and procedure key (‘Operationen- und Prozedurenschlüssel’)
